# Effect of Intrapartum Maternal Hemoglobin on Mode of Delivery and Short-Term Neonatal Outcome: A Systematic Review

**DOI:** 10.1097/OGX.0000000000001074

**Published:** 2022-10-06

**Authors:** Julia Sandra Smith, Lauren Maria Bullens, Marieke Beatrijs van der Hout-van der Jagt, Pieter Jurjen van Runnard Heimel, Swan Gied Oei

**Affiliations:** ∗ Medical Student, Faculty of Health, Medicine, and Life Sciences, Maastricht University, Maastricht, the Netherlands; † Gynecologist, Department of Obstetrics and Gynecology, Streekziekenhuis Koningin Beatrix, Winterswijk, the Netherlands; ‡ Clinical Researcher, Department of Gynecology and Obstetrics, Màxima Medical Center, Veldhoven, the Netherlands; § Biomedical Engineer, Department of Biomedical Engineering, Eindhoven University of Technology, Eindhoven, the Netherlands; ¶ Gynecologist-Perinatologist, Department of Gynecology and Obstetrics, Màxima Medical Center, Veldhoven, the Netherlands; ∥ Professor, Department of Electrical Engineering, Eindhoven University of Technology, Eindhoven, the Netherlands

## Abstract

**Importance:**

Maternal anemia in pregnancy is a common condition worldwide and is considered a risk factor for adverse neonatal and maternal outcome. Also high hemoglobin (Hb) levels are associated with adverse pregnancy outcomes. However, studies regarding the influence of intrapartum maternal Hb on mode of delivery and short-term neonatal outcome are limited and contradicting.

**Objective:**

The aim of this study was to provide an overview of current evidence regarding associations between intrapartum maternal Hb levels and mode of delivery and short-term neonatal outcome. In addition, we propose directions for future research.

**Evidence Acquisition:**

We systematically searched the electronic PubMed, EMBASE, and Cochrane databases for studies on maternal Hb levels and mode of delivery maternal and short-term neonatal outcome until January 2021. Eligible articles and their references were independently reviewed by 2 authors. Assessment was based on methodological quality and study results.

**Results:**

We included 14 studies that evaluated the level of maternal pH in relation to clinical outcome, 6 studies on mode of delivery, 10 studies on Apgar score, 1 study on fetal distress, 2 studies on neonatal intensive care unit admission, 1 study on umbilical cord pH, and 5 studies on perinatal mortality.

**Conclusions and Relevance:**

We found a trend toward an increased risk of cesarean delivery in anemic woman. Concerning the short-term neonatal outcomes, the evidence is conflicting, and included studies are too heterogenic to compare. Furthermore, various studies indicated a relation between high Hb levels and increased perinatal mortality. Therefore, we especially recommend attention to elevated Hb levels.

**Target Audience:**

Obstetricians and gynecologists, family physicians.

**Learning Objectives:**

After completing this activity, the learner should be better able to describe how Hb levels affect mode of delivery and short-term neonatal outcome, and identify abnormal Hb levels and propose appropriate treatment and monitoring recommendations.

During pregnancy, maternal hemoglobin (Hb) concentration drops physiologically due to hemodilution. This effect reaches a maximum in the third trimester.^1,2^ Therefore, the World Health Organization set the cutoff for anemia in pregnancy to Hb <11 g/dL, instead of <12 g/dL in nonpregnant women.^3^ Still, anemia in pregnancy is common; in 2011, the Nutrition Impact Model Study estimated that 38% of pregnant women worldwide are anemic, with iron deficiency as the major cause (75%).^4,5^ Other causes of anemia are folate, vitamin B_12_ and vitamin A deficiencies, chronic inflammation, parasitic infections, and inherited disorders.^5^ Various studies reported on the consequences of anemia in pregnancy.^5–15^ It is thought that low maternal Hb concentration is a risk factor for adverse neonatal and maternal outcomes.^5–15^ A systematic review and meta-analysis reported a higher risk of preterm birth in case of maternal anemia in the first or second trimester,^7^ whereas a more recent meta-analysis showed also an increased risk of low birth weight.^8^

Apart from low Hb, also elevated Hb levels are associated with adverse perinatal outcome.^4,15–17^ As a result of poor plasma expansion, blood viscosity increases, leading to a reduction in blood flow and fetomaternal exchange of oxygen and nutrients in the placenta.^15,16^ High Hb concentrations are associated with pregnancy-induced hypertension and preeclampsia.^15–17^

Because low and elevated Hb levels seem to negatively influence pregnancy outcome, this may indicate a U-shaped optimum for Hb concentration in pregnancy.^14,15,18^ We hypothesize that in both anemic women and women with elevated Hb levels, there is a suboptimal oxygen supply to the placenta. This “placental hypoxemia” may lead to impaired fetal oxygenation, thus increasing the risk of fetal distress and possibly leading to impaired neonatal outcome. Furthermore, in case of anemia, maternal endurance during labor may be impaired, thus increasing the risk of assisted vaginal delivery or cesarean delivery (CD).

Until now, no systematic review has evaluated the relation among maternal Hb concentration, mode of delivery, and neonatal outcome. Individual studies reporting on the course of labor and short-term neonatal outcome in relation to maternal Hb show different results.^10–12,19,20^ Therefore, we aim to investigate the effect of maternal Hb in the second or third trimester of pregnancy on mode of delivery, Apgar score, umbilical cord pH, neonatal intensive care unit (NICU) admission, and perinatal mortality.

## METHODS

### Data Sources

We systematically searched the electronic databases PubMed, EMBASE, and Cochrane for studies that reported on the relationship between maternal Hb and mode of delivery and/or neonatal outcome. The search terms included “h(a)emoglobin,” “h(a)ematocrit,” “mode of delivery,” “f(o)etal distress,” “pregnancy outcome,” “term birth,” and “childbirth.” This search was performed with the help of an information specialist. The study had to be available in the English or Dutch language. In addition, we manually reviewed the list of references of the identified articles and systematic reviews for additional eligible studies that were not identified in the initial search.

### Inclusion and Exclusion Criteria

We included all studies that reported on both the maternal Hb concentration and at least 1 of the following outcome measures: mode of delivery, Apgar score, umbilical cord pH, NICU admission, fetal distress, or perinatal death. The study population had to consist of women with a singleton pregnancy, term birth, and with the intention of a spontaneous vaginal delivery. The maternal Hb concentration had to be measured during the second or third trimester of pregnancy, so it would reasonably reflect the peripartum Hb concentration.^21^ Exclusion criteria were planned CD, multiple pregnancies, or Hb concentration measured in the first trimester only.

### Study Selection

Two independent investigators (J.S. and L.B.) screened all titles and abstracts of trials found in our search to determine if they met the inclusion criteria. Disagreements were discussed and consensus was reached. After eliminating noncompliant articles, the 2 investigators (J.S. and L.B.) analyzed the full text of the remaining studies to decide on eligibility for inclusion.

### Data Extraction and Risk of Bias Assessment

Methodological quality was assessed from the following items: study type, number of subjects, risk of selection bias, including randomization and blinding (high or low), and description of inclusion and exclusion criteria (complete or incomplete). We used the GRADE instrument to provide an overall judgment of the study quality as described in the GRADE Handbook.^22^ Both reviewers evaluated the quality of eligible studies independently. Data were extracted from full texts, tables, and graphs. Data were entered into Microsoft Excel (Excel for Mac 2011; Microsoft Corporation, Redmond, WA), and the 2 reviewers double-checked accuracy.

### Data Analysis

The systematic review was conducted using the PRISMA guidelines and checklist (2009).^23^ A meta-analysis could not be performed because the included articles show large heterogeneity in study population and study methods. Therefore, we described the results and displayed the evidence in relation to the quality of each study.

## RESULTS

### Data Search

**FIG. 1:**
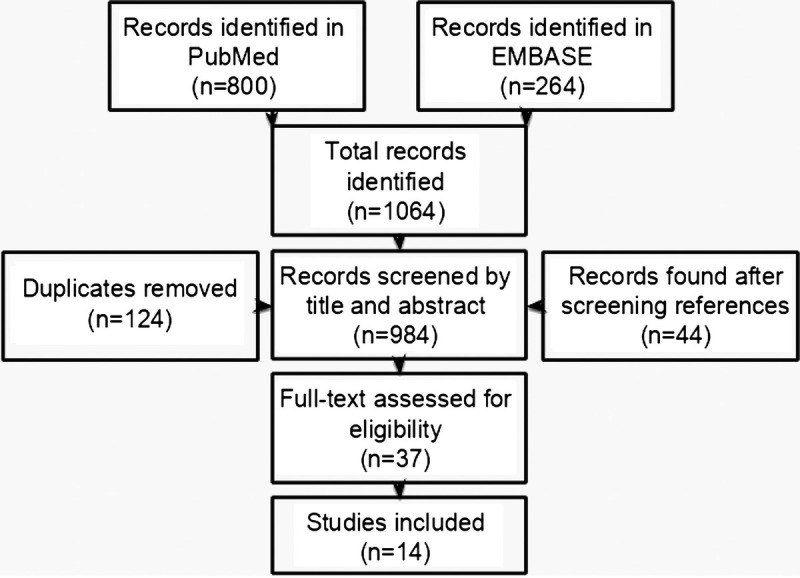
Results from the literature search and the different steps in the selection process of eligible articles.

After removal of duplicates, 940 studies published before January 2021 were found (Fig. 1). The studies were screened for eligibility by title and abstract, and 14 articles were found eligible for full-text assessment. All references were screened, and we found another 44 articles that were screened by title and abstract, of which 23 articles were eligible for full-text assessment. In total, we performed a full-text assessment on 37 articles (Fig. 1). Of these 37 articles, 23 were excluded because of the following reasons: the article was not available in English or Dutch (n = 2); the articles were reviews or meta-analyses (n = 5); the maternal Hb concentrations were only measured in the first trimester or it was not stated in the article when the Hb concentrations were measured (n = 6); no relevant outcome measures were concerned (n = 9); and breech presentations were also included (n = 1). A total of 14 articles, including a total of 422,180 women, met the inclusion criteria. Among the included articles were 6 prospective cohort studies, 2 case-control studies, and 6 retrospective cohort studies (Table 1). The study characteristics and quality assessment are displayed in Table 2, and the outcomes are shown in Tables 3A–F.

**TABLE 1:** Number of Included Studies and Study Type Per Outcome Measure

Outcome Measure	Available Evidence
Mode of delivery	1 prospective cohort studies, 5 retrospective cohort studies
Apgar score	4 prospective cohort studies, 1 prospective case-control study, 4 retrospective cohort studies, 1 retrospective case-control study
Fetal distress	1 retrospective cohort study
NICU admission	2 retrospective cohort studies
Perinatal mortality	1 prospective cohort study, 4 retrospective cohort studies
Umbilical cord pH	1 retrospective cohort study

**TABLE 2:** Characteristics and Quality of Included Studies

Author	Year of Publication	Country of Origin	Study Design	Hb Cutoffs for Anemia	Timing Hb Measurement	Sample Size	Description In/Exclusion	Blinding and Randomization	Risk on Selection Bias	Mode of Delivery	Apgar Score	NICU Admission	Perinatal Mortality	Fetal Distress	Umbilical Cord pHa	Missing Results Reported	Cointerventions Described	Confounding Variables Described	GRADE
Van Bogaert^24^	2006	South Africa	Retrospective cohort study	<10.00 g/dL	Second trimester	3214	Incomplete	NA	High	Yes	No	No	No	No	No	No	Iron and folic acid	No	Low
Orlandini et al^12^	2016	Italy	Retrospective cohort study	<11.00 g/dL	Between AD 35 + 0 and 36 + 6 wk	1131	Complete	NA	High	Yes	Yes	No	No	No	No	No	Multivitamin	Yes	Low
Aimakhu and Olayemi^25^	2003	Nigeria	Prospective cohort study	<9.67 g/dL	Every antenatal visit	633	Incomplete	NA	Moderate	Yes	Yes	No	Yes	No	No	No	Iron	Yes	Low
Fareh et al^26^	2009	United Arab Emirates	Retrospective case-control study	<11.00 g/dL	Around AD 30 wk	200	Complete	NA	High	No	Yes	No	No	No	No	No	No	Yes	Low
Zhang et al^27^	2009	China	Prospective cohort study	<10.00 g/dL	Every trimester	164,667	Incomplete	NA	Moderate	No	No	No	Yes	No	No	No	Iron and folic acid	Yes	Low
Sekhavat et al^28^	2011	Iran	Prospective cohort study	<10.00 g/dL	First stage of labor	18	Incomplete	NA	Moderate	No	Yes	No	No	No	No	No	No	No	Low
Lone et al^29^	2004	Pakistan	Prospective cohort study	<11.00 g/dL	In labor	629	Incomplete	NA	Moderate	No	Yes	No	Yes	No	No	Yes	No	Yes	Low
Drukker et al^19^	2015	Israel	Retrospective cohort study	<11.00 g/dL	In labor	7566	Complete	NA	High	Yes	Yes	Yes	No	No	No	No	No	Yes	Low
Hwang et al^20^	2010	Republic of Korea	Retrospective cohort study	<10.00 g/dL	Third trimester	356	Incomplete	NA	High	Yes	Yes	Yes	Yes	No	No	No	Iron	Yes	Low
Little et al^30^	2004	United Kingdom	Prospective cohort study	<11.00 g/dL	Second trimester	144,209	Incomplete	NA	High	No	No	No	Yes	No	No	No	No	Yes	Low
Lee et al^31^	2006	Korea	Prospective cohort study	<10.8 g/dL	Second trimester	248	Incomplete	NA	Moderate	No	Yes	No	No	No	No	No	No	No	Low
Xiong et al^32^	2003	China	Retrospective cohort study	<10.00 g/dL	AD 32 wk	15943	Incomplete	NA	High	No	No	No	Yes	No	No	No	No	Yes	Low
Lelic et al^33^	2014	Bosnia and Herzegovina	Prospective case-control study	<10.50 g/dL	Second trimester	100	Complete	NA	High	No	Yes	No	No	No	No	No	No	No	Low
Bullens et al^34^	2019	The Netherlands	Retrospective cohort study	<11.0 g/dL	<2 wk prior labor	9144	Complete	NA	Moderate	Yes	Yes	No	No	Yes	Yes	No	No	Yes	Low

GA, gestational age; NA, not applicable.

**TABLE 3:** Outcome of Included Studies

Author	Study Design	Association	Comment
3a Mode of delivery			
Van Bogaert^24^	Retrospective cohort study	Negative	The prevalence of booking anemia (±GA 24 wk) in primigravidas and multigravidas with a CD was significantly higher compared with spontaneous vaginal deliveries (*P* = 0.002).
Orlandini et al^12^	Retrospective cohort study	Negative	Anemic women showed a significantly higher rate in emergency CD (*P* = 0.006).
Aimakhu and Olayemi^25^	Prospective cohort study	Unclear	37.5% of moderate anemic patients delivered by CD compared with 22.2% of the nonanemic patients, there was no statistical test performed.
Drukker et al^19^	Retrospective cohort study	Negative	Rates of CD were significantly higher among anemic women if they were (grand) multiparas. Anemia was identified as a significant independent risk factor of CD (*P* < 0.001).
Hwang et al^20^	Retrospective cohort study	Negative	Anemic women showed a significantly higher rate of CD for fetal distress compared with nonanemic women (*P* < 0.001).
Bullens et al^34^	Retrospective cohort study	Negative	There was a unique significant contribution of Hb for the prediction of IVD for any reason (*P* = 0.00), IVD for fetal distress (*P* = 0.05), CD for any reason (*P* = 0.01), and CD for nonprogressive labor (*P* = 0.01).
3b Apgar score			
Orlandini et al^12^	Retrospective cohort study	No	No differences were observed in terms of Apgar scores at 5 minutes between anemic women and nonanemic women.
Aimakhu and Olayemi^25^	Prospective cohort study	Moderate	Neonates of nonanemic mothers had a higher Apgar score at 1 minute (*P* < 0.05) compared with anemic mothers. There was no significant difference in Apgar score at 5 minutes between the anemic and nonanemic groups.
Fareh et al^26^	Retrospective case-control study	No	There was no significant difference in Apgar score between the case group and control group.
Sekhavat et al^28^	Prospective cohort study	Negative	The risk of low Apgar score was significantly increased in women with anemia. However, the given *P* value given is *P* = 0.8, which indicates no significant association. Therefore, we interpret no significant difference.
Lone et al^29^	Prospective cohort study	Negative	Multivariate analysis showed that the risk of an Apgar score <5 at 1 minute was 1.8 times higher for anemic women compared with nonanemic women.
Drukker et al^19^	Retrospective cohort study	Negative	Anemia was identified as a significant independent risk factor of Apgar score <7 at 5 minutes (*P* < 0.001).
Hwang et al^20^	Retrospective cohort study	No	There was no significant difference in Apgar scores found.
Lee et al^31^	Prospective cohort study	Negative	Anemic women had significantly lower Apgar scores at 1 and 5 minutes compared with nonanemic women (*P* < 0.05).
Lelic et al^33^	Prospective case-control study	No	There was no significant difference in Apgar score between the case group and control group.
Bullens et al^34^	Retrospective cohort	No	There was no relation between Hb level and low Apgar score.
3c Fetal distress			
Bullens et al^34^	Retrospective cohort study	No	Hb levels did not have a significant unique contribution to the likelihood of fetal distress.
3d Neonatal intensive care unit admission			
Drukker et al^19^	Retrospective cohort study	Moderate	Anemia was identified as a significant independent risk factor for NICU admission (*P* = 0.018). When anemia was distributed by severity (mild, moderate/severe), there was no significant difference found between study groups in NICU admission.
	Retrospective cohort study	No	There was no significant difference in NICU admission between groups.
3e Perinatal mortality			
Aimakhu and Olayemi^25^	Prospective cohort study	Unclear	97.4% of the nonanemic patients had live births compared with 75% of the moderately anemic patients and 100% of mild anemic patients. There were no statistical tests performed.
Zhang et al^27^	Prospective cohort study	Moderate	No association among maternal anemia, intrapartum stillbirth, and neonatal mortality was detected. In fact, a trend toward a slightly increased risk of early neonatal death in relation to elevated Hb levels in the second and third trimester was found (hazards ratio, 1.1; 95% CI, 1.0–1.2).
Lone et al^29^	Prospective cohort study	No	The adjusted relative risk of perinatal mortality was 3.2 times higher among anemic women; this was not significant (95% CI, 0.7–14.6).
Little et al^30^	Prospective cohort study	No	A U-shaped pattern was found with lowest recorded Hb concentration (in most cases between GA 26 and 28 wk) for early neonatal mortality; however, when adjusted for prematurity, the relationship of early neonatal mortality with lowest Hb largely disappeared and was no longer significant.
Xiong et al^32^	Retrospective cohort study	No	Anemia in the third trimester was not associated with poor birth outcomes. When divided into different degrees of anemia, there were no significant differences found in the frequency of perinatal mortality.
3f Umbilical cord pH			
Bullens et al^34^	Retrospective cohort study	No	Before correction for confounders (age, parity, and birth weight), there was a significant correlation between Hb levels and umbilical cord pH (≤7.05 of >7.05). After correction, Hb level did not contribute to the prediction of low umbilical cord pH ≤7.05.

### Mode of Delivery

We identified 6 articles reporting on maternal Hb concentration and mode of delivery.^12,19,20,24,25,34^ Van Bogaert^24^ performed a retrospective cohort study in a rural hospital in South Africa. Of the included 3214 patients, 2707 patients had a spontaneous vaginal delivery and a total of 507 patients had a secondary CD. The prevalence of anemia in patients with a CD was significantly higher compared with spontaneous deliveries (odds ratio [OR], 0.55; 95% confidence interval [CI], 0.37–0.80; *P* = 0.002). Unfortunately, they did not describe the reason to perform a CD (eg, fetal distress or nonprogressive labor). Orlandini et al^12^ performed a retrospective cohort study and included 1131 women with uncomplicated pregnancies. There were 2 groups, group A (n = 156) with Hb concentrations <11 g/dL (mild anemia) and group B with Hb ≥11.1 g/dL. Anemic women showed a higher rate of emergency CD than nonanemic women (*P* = 0.006). Aimakhu and Olayemi^25^ performed a prospective cohort study in a University College Hospital in Nigeria. With a finger prick, the packed cell volume (PCV) was measured every antenatal visit until delivery. Hb can be estimated by dividing the PVC by 3,^35^ a PVC below 30% was considered anemic. There were more CD in the moderate anemic group (37.5%), compared with the nonanemic and mildly anemic group (respectively, 22.2% and 21.4%). There were no statistical tests performed because of the small sample size of moderately anemic women (n = 24). Drukker et al^19^ conducted a large retrospective cohort study containing 75,660 women; the Hb values were determined on the day of labor. Maternal anemia was significantly associated with higher rates of CD in multiparous women (2.6% vs 2.1% for multiparas [*P* = 0.039] and 3.2% vs 2.0% for grand multiparas [defined as >5 childbirths] [*P* < 0.001]). There was no significant difference among nulliparous women. In addition, they showed that an increase in Hb levels of 1.0 g/dL was associated with a reduction of 8.3% in CD rate (OR, 0.92; 95% CI, 0.88–0.95; *P* < 0.001). Two stepwise backward logistic regression models were performed to evaluate the independent effect of anemia on CD rate. Both models identified anemia as a significant independent risk of CD (OR, 1.30; 95% CI, 1.13–1.49; *P* < 0.001; and OR, 1.56; 95% CI, 1.23–1.97; *P* < 0.001). Hwang et al^20^ performed a retrospective cohort study as well, including 3560 women of whom 377 had anemia. In the anemic group, there were higher rates of CD for fetal distress compared with the nonanemic group. Also in the multivariate analysis, CD for fetal distress was independently associated with anemia (OR, 1.5; 95% CI, 1.2–1.7; *P* < 0.001). Bullens et al^34^ conducted a large retrospective cohort study in a tertiary hospital in the Netherlands. They included 9144 women where the Hb concentration was determined within 2 weeks before labor. The mean Hb concentration was 12.2 g/dL (±1.2, 7.7–16.4). Intrapartum Hb concentration was low in 12.7%, normal in 60.4%, and high in 26.9%. No women had severe anemia.

Hemoglobin levels were significantly different in the groups where women had an instrumental vaginal (IVD) for fetal distress (*P* < 0.001), IVD for nonprogressive labor (*P* < 0.001), or a CD for nonprogressive labor (*P* < 0.001), compared with the women where these specific assisted-delivery measures were not undertaken.

The absolute difference in mean Hb level was 0.1–0.5 g/dL, and the effect size was small (range Cohen d, 0.01–0.03). Logistic regression was performed to correct for the confounders age, parity, and birth weight. There was a unique significant contribution of Hb for the prediction of IVD for any reason (OR, 1.10; 95% CI, 1.04–1.17; *P* < 0.05), IVD for fetal distress (OR, 1.10; 95% CI, 1.00–1.21; *P* = 0.05), CD for any reason (OR, 0.91; 95% CI, 0.84–0.98; *P* = 0.01), and CD for nonprogressive labor (OR, 0.89; 95% CI, 0.81–0.97; *P* = 0.01).

### Apgar Score

Ten articles reporting on Apgar score were included.^12,19,20,25,26,28,29,31,33,34^ Orlandini et al^12^ did not observe any differences in 5-minute Apgar score between the anemic and nonanemic group. The study by Aimakhu and Olayemi^25^ reported a significantly higher mean 1-minute Apgar scores in the nonanemic group compared with the moderate anemic group. The mean 1-minute Apgar score was 7.9 in the nonanemic group (n = 567), 7.8 in the mild anemia group (n = 42), and 6.4 in the moderate anemia group (n = 24) (*P* < 0.05). However, the mean 5-minute Apgar was not significantly different; the mean Apgar scores for nonanemic, mild, and moderate anemia were, respectively 9.5, 9.6, and 8.6. Fareh et al^26^ performed a retrospective case-control study. Records of 100 consecutive anemic mothers who received antenatal care and had a vaginal delivery in the hospital were reviewed. Within 1 week of the delivery, a nonanemic patient was enrolled for inclusion in the control group. There were no statistically significant differences in baseline characteristics between case and control groups. Apgar scores at 1 and 5 minutes after birth were not different between the 2 groups. Sekhavat et al^28^ performed a prospective cohort study in Iran. A total of 1842 patients fulfilled inclusion criteria of whom 328 patients had anemia and 598 patients had high Hb concentrations. The authors state that the risk of low Apgar score was significantly increased in women with anemia; however, in the results table, a *P* value of 0.8 is mentioned. It is also unclear whether the 1- or 5-minute Apgar score is considered in their study. Lone et al^29^ performed a prospective cohort study and included 629 women. The univariate analysis showed that the risk of a 1-minute Apgar score <5 and 5-minute Apgar score <7 was 2.1 (95% CI, 1.2–3.7) and 1.7 (95% CI, 1.0–3.1), respectively. The multivariate analysis showed that the risk of a low 1-minute Apgar score was 1.8 times higher for anemic women compared with nonanemic women (95% CI, 1.2–3.7). The study of Drukker et al^19^ observed a significantly higher incidence of a 5-minute Apgar score <7 (*P* < 0.001). In the multivariate logistic stepwise regression model, anemia was an independent risk factor for 5-minute Apgar score <7 (OR, 2.21; 95% CI, 1.84–2.64; *P* < 0.001). When a multivariate logistic stepwise regression was performed by the degree of anemia, it showed that women with moderate or severe anemia had significantly increased risks for a low 5-minute Apgar score, compared with women with mild or no anemia (OR, 2.98; 95% CI, 2.20–4.03; *P* < 0.001).

Hwang et al^20^ performed a retrospective cohort study. They did not find any significant differences among study groups for 5-minute Apgar score <7. Lee et al^31^ performed a prospective cohort study with 248 healthy pregnant women. The Hb concentration was measured at 24 to 28 weeks of gestation, and the women were divided into 3 groups: Hb <10.8 g/dL (anemia), Hb 10.8–11.9 g/dL (normal), and Hb ≥12.0 g/dL (high). Newborn infants from anemic mothers had significantly lower Apgar scores at 1 and 5 minutes than the normal and high Hb groups (*P* < 0.05). They also found a significantly positive correlation between maternal Hb concentration and Apgar scores at 1 minute (*r* = 0.231, *P* < 0.05) and at 5 minutes (*r* = 0.201, *P* < 0.05). Lelic et al^33^ performed a prospective case-control study with 2 groups, each consisting of 50 women with healthy term pregnancies. The control group contained women with neither signs of anemia nor any other pregnancy disorder that could affect pregnancy outcomes. The Apgar scores at 1 and 5 minutes in both groups were similar. The study by Bullens et al^34^ did not show a correlation between Hb levels and Apgar scores after correction for confounders.

### Fetal Distress

Bullens et al^34^ reported on (suspected) fetal distress in relation to intrapartum Hb levels. Fetal distress was diagnosed as the suspicion of fetal hypoxia, based on an abnormal fetal heart rate pattern according to the modified FIGO criteria,^36^ fetal scalp blood samples pH <7.20, or significant ST event. In a total of 21.6% of the deliveries, fetal distress was diagnosed. Initially, Hb levels were found to be significantly different among the groups where fetal distress was present or absent. However, after correction for confounders, Hb did not have a significant contribution to the likelihood of fetal distress (*P* = 0.37).

### NICU Admission

We included 2 articles investigating the risk of NICU admission in relation to maternal Hb.^19,20^ Drukker et al^19^ showed that NICU admission occurred more often in anemic mothers (*P* < 0.001). They also concluded that anemia was an independent risk factor for NICU admission (OR, 1.28; 95% CI, 1.04–1.57; *P* = 0.018). However, when distributed by the degree of anemia, there was no significant difference in NICU admission per study group (mild anemia: OR, 1.23; 95% CI, 0.98–1.56 and moderate/severe anemia: OR, 1.45; 95% CI, 0.99–2.13). Hwang et al^20^ did not show any differences in NICU admission among study groups.

### Perinatal Mortality

Five articles on perinatal mortality were included.^25,27,29,30,32^ A Nigerian study conducted in a university hospital reported a perinatal mortality rate of 33 per 1000 births.^25^^25^ Of the nonanemic patients, 97.4% had a live birth, compared with 100% of the mild anemic patients and 75% of the moderately anemic patients. No statistical tests were performed.

Zhang et al^27^ performed a large prospective cohort study in China. A total of 153,952 women were included in the study. The overall perinatal mortality rate was 14.3 per 1000 births. No association between maternal anemia and intrapartum stillbirth or neonatal mortality was detected. In fact, a trend toward a slightly increased risk of early neonatal death in relation to elevated Hb concentrations in the second and third trimester was found. This study used Hb as a continuous value. The authors found a marginally significant association for Hb ≥12 g/dL in the third trimester (hazards ratio, 1.1; 95% CI, 1.0–1.2). Lone et al^29^ reported a 3.2 times higher, but not statistically different risk of perinatal mortality among anemic women (adjusted relative risk, 3.2; 95% CI, 0.7–14.6). Little et al^30^ performed a prospective cohort study, using data of 144,209 pregnancies. There were 903 perinatal deaths in total, of which 689 stillbirths and 214 early neonatal deaths. A U-shaped pattern was found with the lowest recorded Hb concentration (in most cases between gestational age 26 and 28 weeks) for early neonatal mortality rates. However, when adjusted for prematurity, the relationship of early neonatal mortality with the lowest Hb largely disappeared and was no longer significant. Xiong et al^32^ in 2003 performed a retrospective cohort study in China. Their study population consisted of 15,943 women with singleton pregnancies, 95% of the women were primigravidas. Anemia in the third trimester was not associated with a higher risk of perinatal mortality.

### Umbilical Cord pH

The study by Bullens et al^34^ compared the Hb levels of neonates having an umbilical cord pHa ≤7.05 versus >7.05. After correction for confounders, there was no significant correlation with Hb levels.^34^

## DISCUSSION

The aim of this systematic review was to find out if and how intrapartum maternal Hb levels are related to mode of delivery and neonatal outcome.

We selected 14 studies, corresponding to a total of 422,180 patients, and we evaluated the outcome measures: mode of delivery, Apgar score, fetal distress, NICU admission, perinatal death, and umbilical cord pH. We hypothesized that both low and high Hb levels would impair fetomaternal oxygen exchange, potentially leading to fetal distress, assisted or operative delivery, and poor neonatal outcome.

We showed that, for most of the outcome measures, study results were conflicting. Also, included studies showed large heterogeneity in study population, study design, and outcome measurements. As a result, a meta-analysis of the data was not feasible. Because the only limitation for inclusion was language, we believe that the risk of selection bias is low.

### Mode of Delivery

We found 6 studies regarding the outcome measure mode of delivery.^12,19,20,24,25,34^ All studies showed a negative association between low maternal Hb levels and CD rate, implying that anemic women have a higher risk of unplanned CD. Although these studies are all of low quality according to GRADE (Table 2),^22^ they indicate a relation in the same direction. Drukker et al^19^ and Bullens et al^34^ even found a dose-response relation, where the chance of CD increases with the severity of anemia^19,34^ Bullens et al^34^ also investigated the association between Hb levels and IVD rate. The results show a relatively higher mean Hb in women having an IVD of any reason and for the specific indication of fetal distress. In contrast, a study of Malhotra et al^11^ (not included in this review because it did not meet the inclusion criteria) revealed an increased risk of IVD and an increase in duration of labor in women with severe anemia. An explanation for these different outcomes of both studies could be that Malhotra's study did not make a distinction between the indication for IVD (fetal distress or nonprogressive labor). We found 1 other article that studied the risk of IVD in relation to maternal anemia; they found no difference in the chance of having a spontaneous delivery between anemic and nonanemic women.^37^ In conclusion, it is apparent that anemic women have a higher risk of unplanned CD. The relation between maternal Hb and IVD remains unclear.

### Apgar Score

Ten articles were identified for the relation between Hb level and Apgar score.^12,19,20,25,26,28,29,31,33,34^ Three of these studies concluded that maternal anemia is a risk factor for low 1- and/or 5-minute Apgar score, or leads to a lower mean 5-minute Apgar score compared with the group with normal maternal Hb.^19,25,29^ In contrast, 5 studies did not find any differences in Apgar score between anemic and nonanemic mothers.^12,20,26,33,34^ These contradictory findings are probably due to major differences in study design (retrospective and prospective cohort studies, and case-control studies). Furthermore, the size and characteristics of the study populations are diverse: the number of participants varies from 100 to more than 75,000. Some studies excluded women with known hemoglobinopathies, whereas in other studies, these were included. Studies conducted in malaria endemic countries did not make a difference in etiology of the anemia, so the malaria itself can also possibly cause lower Apgar scores. In placental malaria, there is an accumulation of infected red blood cells, which leads to an inflammatory response. This is associated with adverse perinatal outcomes such as stillbirth, low birth weight, and preterm birth.^38,39^ In addition, different outcome measures are used: 5 studies report the mean Apgar score,^25,28,31,33^ whereas others report the number of participants with an Apgar score <5 after 1 minute^29^ or <7 after 5 minutes.^12,19,20,29,34^ In some studies, 1-minute Apgar score is not reported.^12,19,20,34^ Finally, a different definition of anemia is used, with either a Hb cutoff varying between <10 and <11 g/dL,^12,19,20,26,28,29,31,33,34^ or PCV.^25^ Hence, we found conflicting evidence regarding the hypothesis of intrapartum anemia leading to lower Apgar score. As a result, we conclude that anemia may be a risk a factor for low Apgar score.

### Fetal Distress

We only found 1 study reporting on fetal distress, and it showed no relation between Hb level and risk of fetal distress.^34^ Therefore, we have to conclude that there is too little research available to suggest an association.

### NICU Admission

We identified 2 studies that had NICU admission as an outcome measure.^19,20^ Both studies did not found significant difference in NICU admission in anemic woman. However, the study by Drukker et al^19^ did show a trend toward higher NICU admission for children from anemic mothers compared with nonanemic mothers.

This may be explained because the incidence of NICU admission is low (1.7%–2.4%). Thus, when the study group is smaller, differences cannot be longer demonstrated. In conclusion, we cannot confirm a higher risk of NICU admissions in the presence of maternal anemia.

### Perinatal Mortality

Four prospective studies and 1 retrospective study reporting on perinatal mortality were included in this review.^25,27,29,30,32^ None of the studies found a significant association between anemia and perinatal mortality. Zhang et al^27^ demonstrated that high Hb values might be associated with an increased risk of perinatal mortality. Little et al^30^ also showed a U-shaped pattern for Hb concentration and perinatal mortality, but when adjusted for birth weight and prematurity, the association disappeared. No other study included in this review focused on high Hb concentrations and perinatal mortality. However, other studies that did not meet our inclusion criteria also showed a relationship between high Hb and perinatal mortality.^6,18^ An explanation for the association between high Hb and perinatal death may be the poor physiologic adaptation to pregnancy, as a result of placental dysfunction.

The high Hb levels are often caused by failure of normal plasma expansion, leading to increased blood viscosity.^40–44^ As a result of poor plasma expansion or increased blood viscosity, blood flow and fetomaternal exchange of oxygen and nutrients in the placenta are reduced.^40–44^

A systematic review and meta-analysis performed in 2000 concluded that “the relationship between anemia and perinatal mortality was still inconclusive.”^7^ Our study does not indicate otherwise. However, high Hb concentrations should get attention, because of its relation with impaired placental function, potentially leading to an increased risk of perinatal death.^18^

### Umbilical Cord pH

One study used umbilical cord pH as an outcome measure and did not find a significant relationship with Hb levels.^34^ More research is needed to form a conclusion.

### Implications for Clinical Practice

Based on this review, we should prevent peripartum anemia to optimize the chance of a spontaneous delivery and reduce the chance of an unplanned CD. Because the main cause of anemia in pregnancy is iron deficiency, we strongly recommend to monitor Hb levels and when necessary replenish iron stores to correct maternal anemia.^45^ First-line therapy remains oral iron supplementation. Oral iron is readily available, and there is no strong evidence to suggest that intravenous iron therapy is superior to oral iron supplementation in reducing adverse maternal and fetal outcomes. However, intravenous iron should be considered in cases of poor adherence, intolerance to oral iron therapy, or treatment of severe anemia.^46–48^ In developing countries, also parasitic diseases such as malaria are a major cause of maternal anemia.^3^ Apart from the risks associated with malaria-related anemia, infection of the placenta itself also contributes to a higher risk of adverse neonatal outcome.^45^ Also the presence of elevated Hb level is likely to influence maternal and neonatal outcome. In this case, attention should be payed regarding the development of a hypertensive disorder of pregnancy, as this is associated with adverse maternal and perinatal outcome.^4,15–17^

## RECOMMENDATIONS FOR FUTURE RESEARCH

For future research, it would be ideal to work with a large population-based database (including low-risk pregnancies), where data such as obstetric and medical history, the course of Hb level during pregnancy, obstetric complications, and socioeconomic status are carefully stored. In the Netherlands, this could be included in the national database called Perined.^49^ An advantage of administrative data sets is that they are typically very large and cover wide periods that are not achievable financially of logistically through any survey method.^50^ Also, large databases provide more reliable information on outcome measures with a low incidence. Once a large, complete, and reliable database is available, the relationship between various obstetric parameters can be investigated. For example, with population-wide data, the relation between maternal Hb and perinatal outcome and mode of delivery can be identified, including correction for possible confounders. Difficulties in creating a large administrative database include warranting confidentiality and assuring good quality of data.^50^ However, we think the advantages outweigh the disadvantages, and therefore we promote the use of large, reliable databases for research purposes.

## CONCLUSIONS

The findings from our review suggest that maternal anemia during labor contributes to an increased risk of CD. However, evidence regarding the relationship among anemia and fetal distress, low Apgar score, low umbilical cord pH, and risk of NICU admission or perinatal death is contradictory and not conclusive.
